# *Frasier*'s Niles Crane—Sports Psychiatrist? A Response to “Sports Psychiatry: An Update and the Emerging Role of the Sports Psychiatrist on the Sports Medicine Team”

**DOI:** 10.1097/JSM.0000000000001091

**Published:** 2022-11-22

**Authors:** Alexander Smith, Michael Liebrenz

**Affiliations:** Department of Forensic Psychiatry, University of Bern, Bern, Switzerland

***Dear Editor-in-Chief:***

We applaud Glick et al.'s article, which describes developments in sports psychiatry, identifies future research, and recommends integrating mental health experts across various disciplines.^1^ The authors adeptly summarize this fast-moving field.

Interestingly, an episode of the popular US sitcom, *Frasier*, dramatizes several of Glick et al's discussions. *Frasier* follows the life of eponymous character, Frasier Crane, a psychiatrist turned radio host. In “Head Game” (1996), Frasier's brother, Niles Crane (also a psychiatrist), encounters a struggling Seattle Sonics' basketballer, Reggie McLemore, while deputizing on the radio show.^2^

Once “unstoppable,” Reggie's form has suffered: “Because of me we have lost six in a row.” Accordingly, he consults with Niles, who conducts various psychotherapeutic exercises, including positive visualization. Subsequently, Reggie's competitive levels improve and he scores match-winning points. This provokes an amusing exchange between Niles and his father: “You turned Reggie's game around in only two minutes?” “You could be […] less surprised. I am a skilled psychiatrist!”

Nonetheless, for Reggie, this improved performance is solely because of a newfound superstition (namely, rubbing Niles' hair), rather than any psychological intervention. The episode thus ends in slapstick impasse, with Niles affirming Reggie's need for long-term therapy and Reggie requesting scissors for Niles' hair.

In our view, “Head Game” encompasses pertinent themes illustrated by Glick et al. For example, Niles embodies sports psychiatry's evolving clinical effects, from caregiving to performance enhancing. Disregarding the effectiveness of psychotherapy, Reggie exhibits enduring stigmas, revealing a lack of knowledge about psychological support. Moreover, the ending exemplifies challenges in shaping entrenched attitudes about psychiatry in a sporting environment.

Albeit a developing discipline, sports psychiatry has frequent depictions in popular culture. We believe “Head Game” provides another relevant (if comedically exaggerated) example. Such representations encourage increasing societal awareness towards sports psychiatry, raising hopes of economic concessions for athletes' mental health care—another consideration that Glick et al. highlight.
